# Real-world use and impact of direct oral anticoagulants among atrial fibrillation patients with cardioembolic stroke

**DOI:** 10.3389/fneur.2026.1844341

**Published:** 2026-06-18

**Authors:** Vladislavs Sokaļskis, Kaspars Kupics, Kristaps Jurjāns, Nikolajs Ņesterovičs, Ieva Ozoliņa, Guntis Karelis, Evija Miglāne, Andrejs Ērglis

**Affiliations:** 1 Clinic of Cardiovascular Diseases, Riga East University Hospital, Riga, Latvia; 2 Latvian Cardiology Center, Paul Stradins Clinical University Hospital, Riga, Latvia; 3 Department of Neurology and Neurosurgery, Pauls Stradins Clinical University Hospital, Riga, Latvia; 4 Department of Neurology and Neurosurgery, Riga East University Hospital, Riga, Latvia; 5 Department of Infectology, Riga Stradins University, Riga, Latvia

**Keywords:** atrial fibrillation, direct oral anticoagulants, non-adherence, real-world evidence, stroke severity

## Abstract

**Background and aims:**

Atrial fibrillation (AF) is a primary cause of cardioembolic stroke (CES). Non-adherence to direct oral anticoagulants (DOACs) remains a major health policy issue. This study aimed to investigate real-world DOAC usage among AF patients presenting with stroke and to evaluate the impact of prior DOAC therapy on stroke severity.

**Methods:**

This retrospective study analyzed data on DOAC usage from CES patients with AF admitted to the two largest Latvian university hospitals between January 2022 and December 2024. We further compared patients’ characteristics receiving prior DOAC therapy and those who were not, confirming our findings by a propensity-matched analysis of both groups.

**Results:**

Of the 2,610 CES patients enrolled, the largest subgroup (43.4%) consisted of individuals with known AF who were not on anticoagulation therapy before the stroke event. A propensity-matched analysis adjusted for demographics, arrival time, AF type, HAS-BLED, and CHA_2_DS_2_-VA score (352 patients per group) revealed that patients taking DOACs presented with significantly milder strokes than non-users (mean admission NIHSS score: 8.84 ± 6.76 vs. 11.58 ± 6.98, *p* < 0.001). Stroke patients on DOAC therapy demonstrated significantly larger left atrial volume indices (LAVI) than non-users (56.05 ± 19.31 vs. 46.94 ± 14.33, respectively; *p* < 0.001).

**Conclusion:**

Non-adherence to DOACs is a substantial contributor to the CES burden. Prior DOAC use is associated with significantly reduced initial stroke severity. Patients with CES while on DOACs exhibited substantially larger left atria.

## Introduction

Atrial fibrillation (AF) is the most common arrhythmia, affecting 1–2% of the general population – a prevalence that has tripled over the last five decades (1, 2). Stroke remains a major complication of AF; non-valvular AF confers a fivefold increased risk of stroke, a risk that escalates further with advancing age (3, 4). Historically, cardioembolic strokes (CES) due to AF have accounted for an estimated 30% of all ischemic events (5). The advent of direct oral anticoagulants (DOACs) revolutionized stroke prevention in AF patients by reducing thromboembolic events while offering a superior safety profile compared to vitamin K antagonists (e.g., warfarin). DOACs present lower risks of severe bleeding and eliminate the need for routine monitoring (6).

The age-adjusted mortality rate related to AF in Europe linearly increased from 12.3 per 100,000 population in 2008 to 15.3 in 2019, reflecting an average annual percentage change of +2.0%. Some Eastern European nations, including Latvia, Lithuania, and Poland, experienced even sharper increases (7), starkly contrasting with the concurrent decreases in mortality rates associated with coronary artery disease (8). As of 2019, the overall stroke incidence in Latvia stood at 183.6 per 100,000 inhabitants (9).

Medication non-adherence might be a critical factor driving these outcomes. Recent data concerning Latvian AF patients demonstrated that 55.8% were not taking DOACs regularly (defined as adherence below 80% of the total intake time), and 30.6% had a gap of ≥30-days in their DOAC use (10). Notably, this is the first Eastern European study to quantify adherence rates utilizing prescription data from a national health registry. In other Eastern European nations, data on adherence to DOACs are restricted to single-center studies, showing appropriate DOAC use prior to stroke ranging from 30% in Romania (2016–2017) (11) to 37% in Poland (2022–2023) (12) and up to 70% in the Czech Republic (2021) (13). Several studies from Western European or North American national health registries showed much better non-adherence rates (ranging from 10 to 24%) (14–17). Adequate adherence to DOACs (defined as a proportion of days covered (PDC) > 80%) is associated with a 31% reduction in ischemic stroke and 14% decrease in all-cause mortality (18). A recent clinical overview highlighted this disparity, noting a widespread lack of studies assessing the direct association between medication non-adherence and clinical outcomes (19).

Numerous studies have observed that ischemic strokes occurring despite anticoagulation therapy tend to be less severe and present with reduced hemorrhagic transformation. This neuroprotective effect is potentially mediated by thrombin inhibition, which stabilizes the blood–brain barrier (20–22). However, prior research has largely analyzed all-comer stroke populations (with or without AF) rather than focusing specifically on AF patients presenting with CES.

Furthermore, preliminary studies have documented enlarged left atria (LA) in anticoagulated stroke patients (23, 24). Ogata et al. demonstrated that increasing LA diameter is predictive of recurrent strokes even in patients receiving anticoagulation (25), suggesting that LA enlargement independently promotes thrombogenicity. To our knowledge, the present study is the first to compare the left atrial volume index (LAVI) in CES patients with AF who are on DOACs vs. those who are not, aiming to generate novel research hypotheses.

Pauls Stradins Clinical University Hospital (PSKUS) and Riga East University Hospital (RAKUS) serve as the primary and secondary medical facilities for stroke treatment in Riga. Located on opposite sides of Daugava River, their combined catchment areas serve approximately 1 million people, or roughly 55% of the Latvian population (26).

A noteworthy strength of the Latvian medical system, similar to those in Estonia and Lithuania, is its electronic interconnectivity. For example, to ensure that the diagnosis of a new-onset AF is correct, a treating doctor can access prior in- and outpatient discharge letters of the other university hospital, access a national archive of medical diagnostic data (i.e., previous ECGs), and see whether any anticoagulants have been prescribed to a patient previously and even whether the patient has obtained them in the pharmacy.

The aims of this retrospective study were: 1. To analyze how recent DOAC adherence data from the national health registry translate into the real-world characteristics of stroke patients in both university hospitals in the years 2022–2024. 2. To evaluate the impact of prior DOAC use on stroke severity, specifically in the CES population, using propensity score matching to adjust for key variables. 3. As a hypothesis-generating exploration, to compare LAVI and LVEF in the CES patients with or without prior DOAC therapy.

## Methods

### Patient population

**Figure 1:**
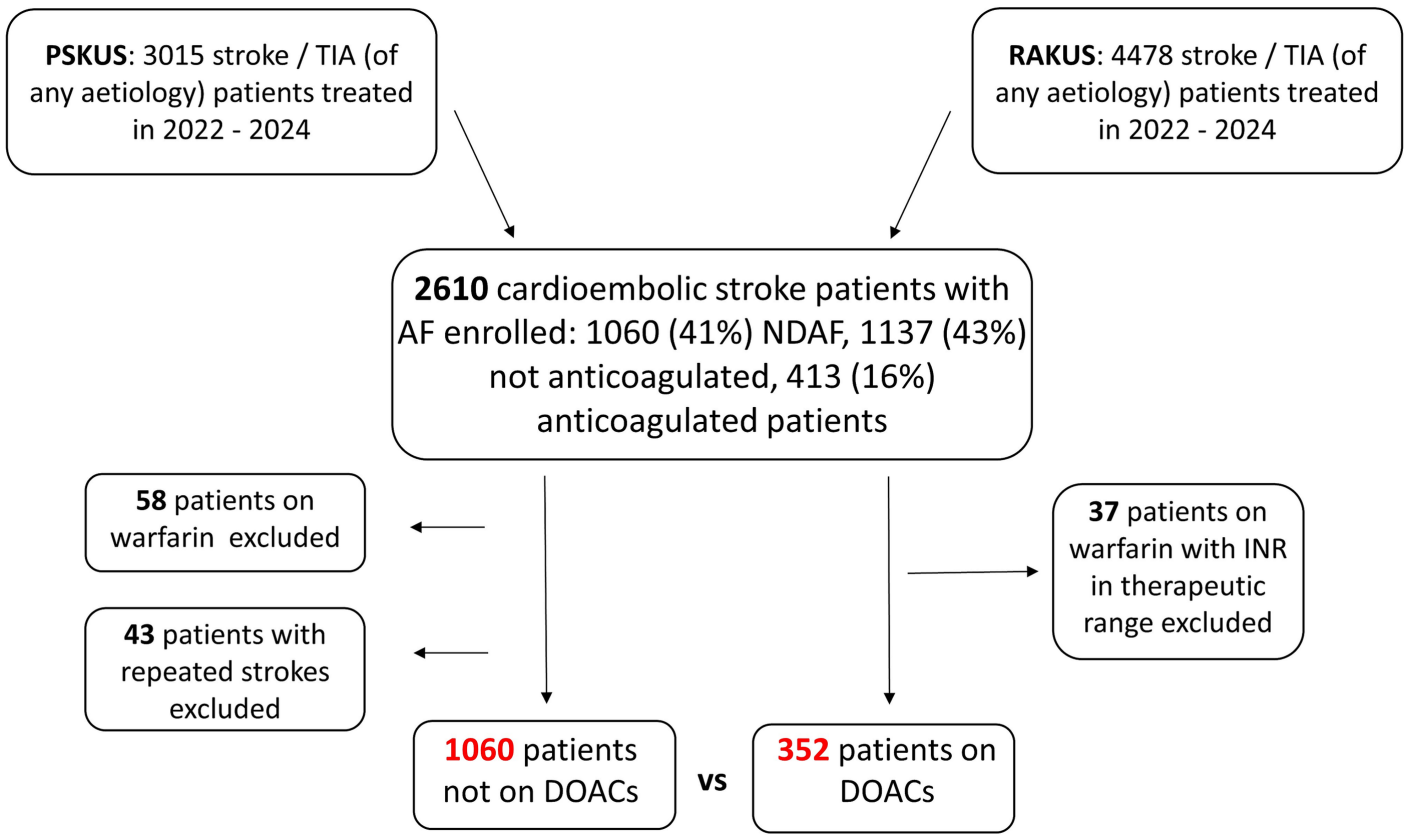
A flowchart of the study design. To assess DOAC usage, we enrolled 2,610 cardioembolic stroke (CES) patients with atrial fibrillation (AF)—i.e., 34.8% of the 7,493 patients presenting with stroke of any etiology at PSKUS and RAKUS during 2022–2024. After excluding patients on warfarin and those with recurrent strokes, we compared 1,060 patients not on direct oral anticoagulants (DOACs) prior to CES with 352 patients on DOACs. AF, atrial fibrillation; CES, cardioembolic stroke; DOACs, direct oral anticoagulants; INR, International Normalized Ratio; NDAF, newly diagnosed atrial fibrillation; PSKUS, Paul Stradins Clinical University Hospital; RAKUS, Riga East University Hospital; TIA, transient ischemic attack.

This retrospective study included AF patients diagnosed with ischemic stroke of cardioembolic origin (including transient ischemic attack, hereafter referred to collectively as CES for brevity). Patients were admitted to the Neurovascular Departments of RAKUS or PSKUS, and diagnoses were made according to the TOAST (Trial of ORG 10172 in Acute Stroke Treatment) criteria (27). The diagnosis was based on CT data, vascular imaging, and prolonged rhythm monitoring. Patients were excluded if they had undergone cardioversion, cardiac intervention, or surgery within the 2 weeks before admission, or if they had a non-stroke-related hospitalization in the previous week, as these factors made the stroke etiology uncertain (58 patients excluded). Patients transferred between the two hospitals for mechanical thrombectomy were attributed to the discharging hospital. For comparative analysis between unique DOAC users and non-users, 43 cases of recurrent stroke were excluded. The study protocol is summarized in Figure 1.

### Co-variates

Demographic factors: age at the time of admission and sex.

Clinical variables: CHA_2_DS_2_-VA and HAS-BLED scores were retrospectively calculated according to the latest ESC guidelines for AF management (28); the incident stroke event was excluded from these baseline calculations.

The AF type was classified as paroxysmal and permanent, with the latter category also including persistent AF forms. A cardioversion of AF was not performed routinely during the hospital stay.

The severity of stroke in this cohort was assessed using a Latvian version of the National Institutes of Health Stroke Scale (NIHSS) (29). To measure the degree of disability or dependence in daily activities, the modified Rankin Scale (mRS) was used.

The arrival time was defined as the time from the onset of stroke symptoms (or the last time a patient was seen healthy) to the first contact with a neurologist in the Emergency Department and is further categorized into ≤ 4.5 h, 4.6 - 9 h, and > 9 h.

Medications prior to admission: Patients were classified as not being on appropriate anticoagulation therapy if they were using only antiplatelet agents, were on warfarin with an INR < 2, or had not taken their DOAC within 48 h before the stroke event. To improve readability, those patients not being on adequate anticoagulation therapy were classified as being “not anticoagulated.” A 48-h window was chosen to obtain robust results as approximately one-third of stroke patients arrived late with stroke symptoms, including those with a so-called wake-up stroke.

An assessment of whether the DOAC dose was adequately reduced was based on the patient’s age, weight, and renal function, according to the threshold per drug label.

Treatments: intravenous thrombolysis, mostly with alteplase, and mechanical thrombectomy.

Laboratory values: Creatinine was measured in μmol/l. The glomerular filtration rate was calculated using the MDRD formula.

Echocardiographic values: We included the echocardiographic data up to 4 months post-stroke event. For this analysis, we included LVEF and LAVI (30).

### Statistical analysis

Descriptive statistics utilized means and standard deviations. Between-group differences were evaluated using the Mann–Whitney U test for continuous variables, and the chi-square test was used for categorical variables. Given the retrospective design and the observed imbalance in baseline clinical complexity, we conducted propensity score matching between DOAC users and non-users based on age, sex, AF type, CHA_2_DS_2_-VA score, HAS-BLED score, and arrival time to mitigate confounding. Subsequent analysis confirmed adequate covariate balance. Statistical significance was established at a *p*-value of < 0.05.

The ethical aspects of this retrospective study were approved by the Ethics Committee of Latvian University (nr. 13–22/90, May 2025) and both local university hospital ethical committees. It was conducted in accordance with the Declaration of Helsinki.

## Results

### General characteristics, assessment of anticoagulation among CES patients with AF

Between January 2022 and December 2024, a total of 2,610 patients were enrolled in this study. The median age was 81 (interquartile range 74–86; min 36, max 102) years, with a male prevalence of 34.0%.

The total number of patients with stroke (and TIA) treated in PSKUS was 1,009 in 2022 (431 patients with CES and AF, or 43% were enrolled). Stroke patients not enrolled (e.g., 578 of 1,009 in PSKUS 2022) had ischemic strokes of non-cardioembolic origin (e.g., large-artery atherosclerosis, small-vessel occlusion/lacunar, or undetermined etiology after extensive evaluation), hemorrhagic strokes, or did not have documented AF. 183 CES patients (out of 431 patients) were diagnosed with new AF. 200 patients (or 46% of all enrolled CES patients with AF) were considered not to be on adequate anticoagulation (AC). Out of 180 patients not on DOAC therapy prior to stroke, 152 patients were not taking DOACs “long-term” and 28 patients for ≤7 days; 20 patients were on warfarin but presented subtherapeutic INR levels. 47 (11%) patients were taking DOACs, and 1 patient was on warfarin with INR in a therapeutic range. Thus, 21 warfarin patients and 3 non-DOAC users with recurrent strokes were excluded from the final analysis of DOAC vs. non-DOAC users.

In 2023, out of 1,017 stroke patients in PSKUS, 421 patients (or 42%) with CES and AF were enrolled in the study. 164 (of 421 enrolled CES patients, i.e., 39%) showed newly diagnosed AF. 187 (44% of the enrolled) patients were not on adequate AC. 139 patients were “long-term” non-DOAC users, and 33 patients were not using them for ≤7 days. 15 patients on warfarin showed subtherapeutic INR. 65 patients were on DOAC therapy, and 5 patients were on warfarin with therapeutic INR. Thus, 20 warfarin patients and 4 non-DOAC users with repeated strokes were excluded.

In 2024, out of 989 stroke patients in PSKUS, 379 patients (or 38%) with CES and AF were enrolled in the study. 159 (of 379 enrolled CES patients, i.e., 42%) showed newly diagnosed AF. 166 (44% of the enrolled) patients were not on adequate AC. 147 patients were “long-term” non-DOAC users, and 15 patients were not using them for ≤7 days. 4 patients on warfarin showed subtherapeutic INR. 51 patients were on DOAC therapy, and 3 patients were on warfarin with therapeutic INR. Thus, 7 warfarin patients and 3 non-DOAC users with repeated strokes were excluded.

In RAKUS, out of 1,454 stroke patients, 457 CES patients (i.e., 31%) with AF were enrolled in 2022. 184 (of 457 enrolled CES patients, i.e., 40%) showed newly diagnosed AF. 196 (43% of the enrolled) patients were not on adequate AC. 158 patients were “long-term” non-DOAC users, and 29 patients were not using DOACs for ≤7 days. 9 patients on warfarin showed subtherapeutic INR. 68 patients were on DOAC therapy, and 9 patients were on warfarin with therapeutic INR. Thus, 18 warfarin patients and 3 non-DOAC users, 4 DOAC users with repeated strokes were excluded.

In 2023, of the 1,539 stroke patients in RAKUS, 504 (or 33%) with CES and AF were enrolled in the study. 196 (of 504 enrolled CES patients, i.e., 39%) showed newly diagnosed AF. 234 (46% of the enrolled) patients were not on adequate AC. 217 patients were “long-term” non-DOAC users, and 13 patients were not using them for ≤7 days. 4 patients on warfarin showed subtherapeutic INR. 62 patients were on DOAC therapy, and 12 patients were on warfarin with therapeutic INR. Thus, 16 warfarin patients and 3 non-DOAC users, 9 DOAC users with repeated strokes were excluded.

In 2024, out of 1,485 stroke patients in RAKUS, 418 CES patients with AF (i.e., 28%) were enrolled. 174 (of 418 enrolled CES patients, i.e., 42%) showed newly diagnosed AF. 154 (37% of the enrolled) patients were not on adequate AC. 119 patients were “long-term” non-DOAC users, and 29 patients were not using them for ≤7 days. 6 patients on warfarin showed subtherapeutic INR. 83 patients were on DOAC therapy, and 7 patients were on warfarin with therapeutic INR. Thus, 13 warfarin patients and 3 non-DOAC users, 11 DOAC users with repeated strokes were excluded.

**Figure 2:**
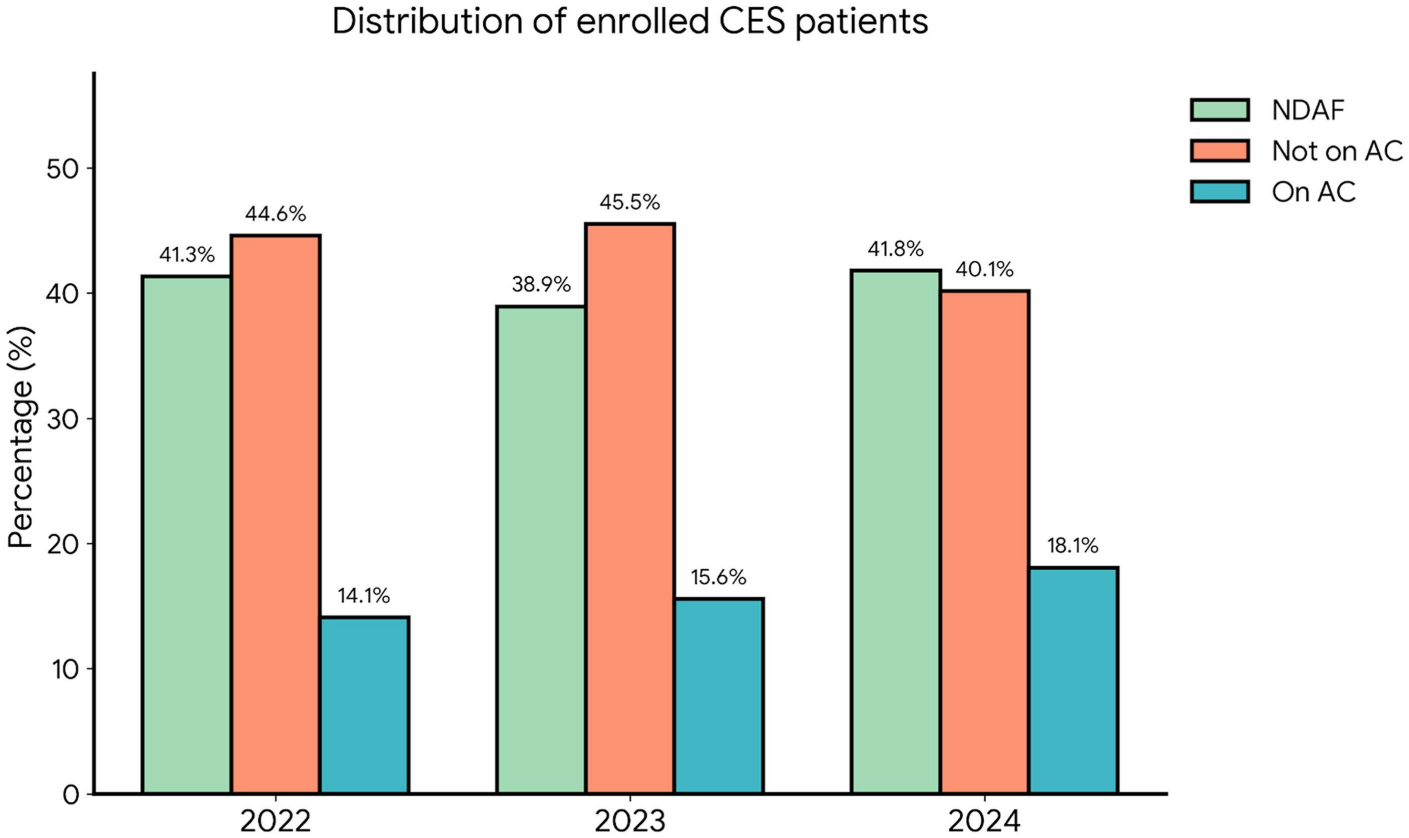
Characteristics of cardioembolic stroke patients from 2022–2024 treated at PSKUS and RAKUS. The graph illustrates that CES patients lacking adequate anticoagulation (AC) constitute (on average) the largest group of cardioembolic stroke patients, representing more than twice the number of patients on AC. A noticeable trend shows a relative increase in the number of patients on AC over the years. Furthermore, 41% of 2,610 CES patients presented with newly diagnosed atrial fibrillation. Although not being on AC, they represent a separate patient cohort, highlighting the importance of timely screening for atrial fibrillation (AF). Percentages are calculated relative to the total CES + AF cohort enrolled in each respective year (2022: *n* = 888; 2023: *n* = 925; 2024: *n* = 797). AC, anticoagulation; AF, atrial fibrillation; CES, cardioembolic stroke; NDAF, newly diagnosed atrial fibrillation; PSKUS, Paul Stradins Clinical University Hospital; RAKUS, Riga East University Hospital.

CES patients with known AF not on anticoagulation treatment, alongside those with newly diagnosed AF, consistently formed the largest cohorts throughout the study period, as shown in Figure 2 and Table 1. However, comparing 2022 to 2024, we observed a relative increase in the proportion of CES patients presenting on anticoagulation (from 14 to 18%) and a corresponding decrease of 76 cases among non-anticoagulated patients.

**Table 1:** Categorization of cardioembolic stroke patients with atrial fibrillation based on anticoagulation status at RAKUS and PSKUS.

	NDAF	NDAF (%)	Not on AC	Not on AC (%)	On AC	On AC (%)	Total
RAKUS							
2022	184	40.26	196	42.89	77	16.85	457
2023	196	38.89	234	46.43	74	14.68	504
2024	174	41.63	154	36.84	90	21.53	418
PSKUS							
2022	183	42.46	200	46.40	48	11.14	431
2023	164	38.95	187	44.42	70	16.63	421
2024	159	41.95	166	43.80	54	14.25	379

Non-anticoagulated cardioembolic stroke patients (43% of the total cohort) formed the largest patient group. Patients with newly diagnosed atrial fibrillation constituted 41%, a similarly large patient group. AC, anticoagulation; NDAF, newly diagnosed atrial fibrillation; PSKUS, Paul Stradins Clinical University Hospital; RAKUS, Riga East University Hospital.

### Characteristics of CES patients with AF not on anticoagulation therapy

Among patients not on anticoagulation therapy prior to CES, the median age of 1,137 patients in this category was 80 years, and 62% were female and 38% were male. Patients on a subtherapeutic dose of warfarin decreased from 29 (7.3%) in 2022 to 10 (3.1%) in 2024.

When comparing 924 patients not using DOACs for >7 days with 136 patients not using DOACs for ≤7 days, a significant difference in admission NIHSS score was observed, although mRS score differences were not significant (NIHSS score: 11.82 ± 7.37 vs. 10.21 ± 6.74, *p* = 0.02; mRS score at admission: 4.21 ± 1.07 vs. 4.07 ± 1.06, *p* = 0.08).

### Characteristics of stroke patients with AF on anticoagulation therapy

413 AF patients admitted with stroke were 68% female and 32% male, and 81 years was the median age. 37 (9.0%) were taking warfarin with their INR at admission ≥2. Among all four available DOACs, the most frequent was rivaroxaban (211 users out of 352, 60.0%), then edoxaban with 106 users (30.0%), 28 patients using dabigatran (8.0%), and 7 apixaban users (2.0%).

143 out of 352 (40.6%) patients were taking the reduced dose of DOACs, and in 88 of 352 cases (25.0%) it was inadequately reduced, likely based on the patient’s age or several comorbidities rather than, for example, their weight or renal function.

### Comparison of DOAC users vs. non-users

We identified 352 patients on DOAC therapy with a median age of 81 years, of whom 32% were male, and compared them against 1,060 patients not receiving DOACs with a median age of 81 years, of whom 34% were male. Supplementary Table 1 provides baseline characteristics and outcomes stratified by 10-year age cohorts for both the non-DOAC and DOAC groups.

DOAC users presented with significantly higher CHA_2_DS_2_-VA scores (4.55 ± 1.43 vs. 4.21 ± 1.46, *p* < 0.0001), driven by higher rates of prior thromboembolic events and congestive heart failure, see Table 2. There was a significant difference in creatinine levels. However, DOAC users experienced significantly milder strokes (NIHSS score at arrival 8.84 ± 6.76 vs. 11.62 ± 7.31, *p* < 0.001) and better functional scores (admission mRS: 3.73 ± 1.20 vs. 4.19 ± 1.07, *p* < 0.001).

**Table 2:** Comparison of baseline characteristics and outcomes between patients on DOAC therapy vs. those not on DOAC therapy.

	Total (n)	No DOAC (*n* = 1,060)	On DOAC (*n* = 352)	*p*-value
Age	1,412	79.14 ± 9.44	79.02 ± 9.23	0.923
Sex	1,412	696F / 364M	239F / 113M	0.442
AF type (parox. / perm.)	1,412	240/820	70/282	0.279
CHA_2_DS_2_-VA Score	**1,412**	**4.21 ± 1.46**	**4.55 ± 1.43**	**< 0.001**
Congestive heart failure	**1,412**	**655**	**243**	**0.014**
Art. hypertension	1,412	897	299	0.914
GFR (ml / min)	**1,411**	**68.6 ± 22.4**	**64.7 ± 21.7**	**0.002**
Diabetes mellitus	1,412	192	74	0.233
Prior stroke / TIA / TE	**1,412**	**338**	**153**	**< 0.001**
Vascular diseases	1,412	285	108	0.169
Arrival time period (1/2 / 3)	1,412	492/108 / 460	181/36 / 135	0.084
NIHSS at arrival	**1,408**	**11.6 ± 7.3**	**8.8 ± 6.8**	**< 0.001**
NIHSS at discharge	**1,209**	**6.6 ± 6.2**	**5.3 ± 5.9**	**< 0.001**
NIHSS difference	**1,209**	**3.5 ± 4.5**	**2.9 ± 4.2**	**0.006**
Thrombolysis	**1,412**	**315**	**12**	**< 0.001**
Thrombectomy	1,412	107	35	0.935
LAVI (ml/m2)	**357**	**47.2 ± 14.8**	**56.1 ± 19.3**	**< 0.001**
LVEF (%)	357	51.7 ± 10.5	52.0 ± 11.1	0.608
HAS-BLED Score	1,412	1.6 ± 0.8	1.6 ± 0.8	0.658
mRS score at arrival	**1,412**	**4.2 ± 1.1**	**3.7 ± 1.2**	**< 0.001**
mRS score at discharge	**1,209**	**3.3 ± 1.4**	**2.9 ± 1.5**	**< 0.001**
mRS score difference	1,209	0.8 ± 1.1	0.8 ± 1.0	0.464

Data are presented as mean ± SD or n (%). AF, atrial fibrillation; DOAC, direct oral anticoagulant; GFR, glomerular filtration rate; LAVI, left atrial volume index; LVEF, left ventricular ejection fraction; mRS, modified Rankin Scale; NIHSS, National Institutes of Health Stroke Scale; TE, thromboembolic event; TIA, transient ischemic attack. Bold values indicate a significant difference (*p* < 0.05).

**Figure 3:**
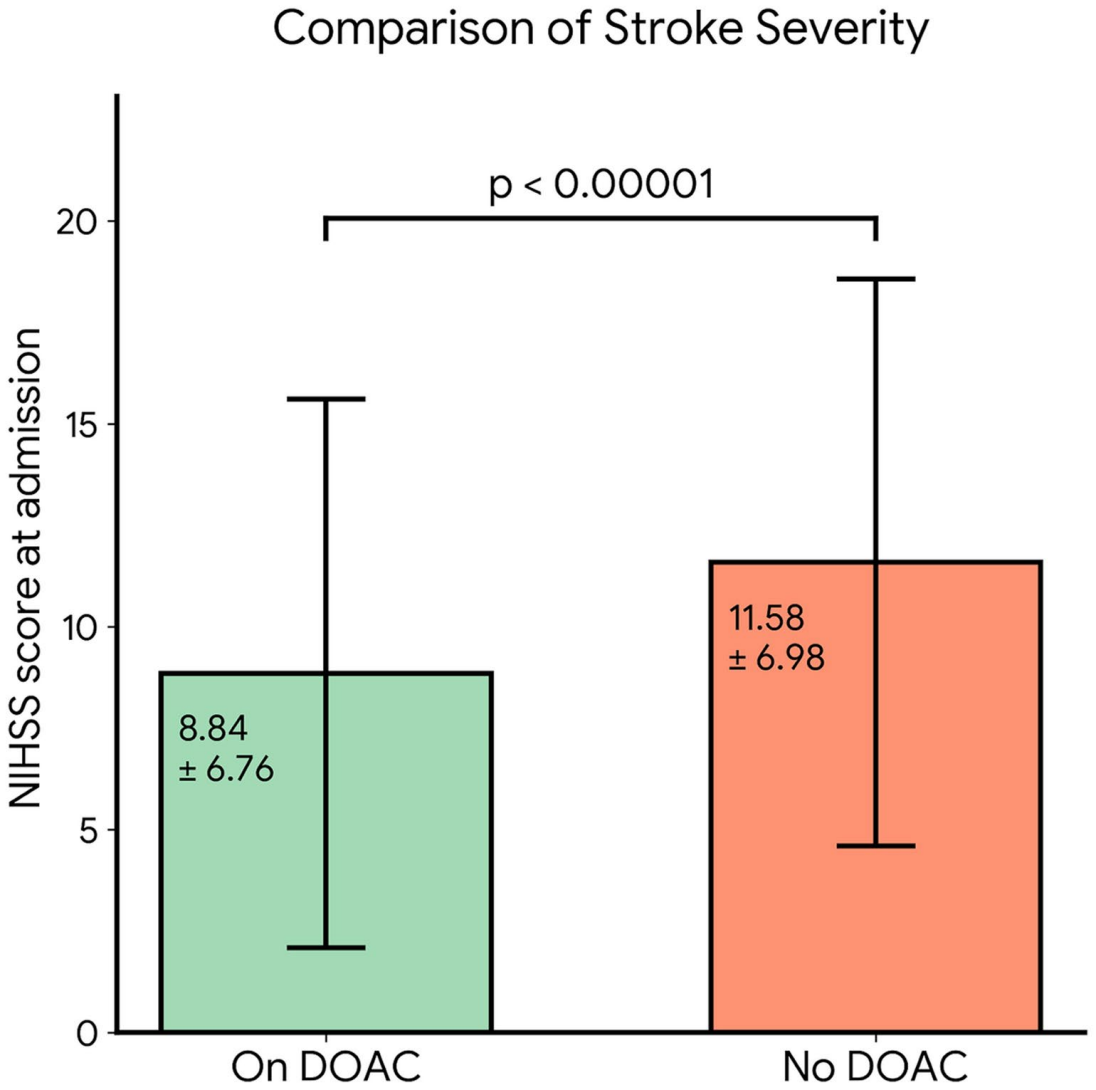
Comparison of stroke severity at arrival between AF patients with and without prior DOAC therapy. Following propensity matching based on age, sex, AF type, arrival time period, HAS-BLED, and CHA_2_DS_2_-VA scores, we compared the initial stroke severity (based on symptoms measured by NIHSS score). Patients lacking prior DOAC therapy presented with significantly more pronounced stroke symptoms compared to DOAC users (11.58 ± 6.98 vs. 8.84 ± 6.76, *p* < 0.001). AF, atrial fibrillation; DOAC, direct oral anticoagulants.

After propensity matching based on the criteria described in the methods section, we repeated the comparison with the results shown in Table 3. The 352 patients in each group differed significantly only regarding the stroke severity at admission (NIHSS score 11.58 ± 6.98 vs. 8.84 ± 6.76 for non-users and DOAC users, respectively, *p* < 0.0001, see Figure 3). Similarly, there was a significant difference in the mRS score (3.73 ± 1.20 vs. 4.20 ± 0.98, *p* < 0.001). There was also a significant difference in the improvement of NIHSS score during the hospital stay, and the frequency of thrombolysis (12 vs. 107, *p* < 0.001).

**Table 3:** Propensity-matched comparison of characteristics and outcomes between patients without prior DOAC therapy and those on DOAC therapy.

	Total (n)	No DOAC (*n* = 352)	On DOAC (*n* = 352)	*p*-value
Age	704	79.0 ± 9.2	78.5 ± 9.2	0.605
Sex	704	236F / 116M	239F / 113M	0.810
AF type (parox. / perm.)	704	77/275	70/282	0.517
CHA_2_DS_2_-VA Score	704	4.4 ± 1.5	4.55 ± 1.43	0.782
Congestive heart failure	704	235	243	0.519
Art. hypertension	704	301	299	0.832
GFR (ml / min)	704	66.8 ± 22.1	64.7 ± 21.7	0.176
Diabetes mellitus	704	80	74	0.686
Prior stroke / TIA / TE	704	260	153	0.077
Vascular diseases	704	111	108	0.807
Arrival time period (1/2 / 3)	703	176/40 / 135	181/36 / 135	0.983
**NIHSS at arrival**	**704**	**11.6 ± 7.0**	**8.8 ± 6.8**	**< 0.001**
**NIHSS at discharge**	**629**	**6.7 ± 6.6**	**5.3 ± 5.9**	**0.001**
**NIHSS difference**	**629**	**3.7 ± 4.5**	**2.9 ± 4.2**	**0.003**
**Thrombolysis**	**704**	**107**	**12**	**< 0.001**
Thrombectomy	704	30	35	0.516
**LAVI (ml/m2)**	**223**	**46.9 ± 14.3**	**56.1 ± 19.3**	**< 0.001**
LVEF (%)	223	51.2 ± 11.3	52.0 ± 11.1	0.568
HAS-BLED Score	704	1.6 ± 0.8	1.6 ± 0.8	0.885
**mRS score at arrival**	**704**	**4.2 ± 1.0**	**3.7 ± 1.2**	**< 0.001**
**mRS score at discharge**	**629**	**3.3 ± 1.4**	**2.9 ± 1.5**	**< 0.001**
mRS score difference	629	0.9 ± 1.0	0.8 ± 1.0	0.464

Groups were propensity-matched based on age, sex, AF type, CHA_2_DS_2_-VA score, HAS-BLED score, and arrival time period. DOAC users presented with significantly less symptomatic stroke, measured by NIHSS score. Patients on DOACs demonstrated a significantly larger LAVI. Data are presented as mean ± SD or n (%). AF, atrial fibrillation; DOAC, direct oral anticoagulant; GFR, glomerular filtration rate; LAVI, left atrial volume index; LVEF, left ventricular ejection fraction; mRS, modified Rankin Scale; NIHSS, National Institutes of Health Stroke Scale; TE, thromboembolic event; TIA, transient ischemic attack. Bold values indicate a significant difference (*p* < 0.05).

A significant difference in LAVI remained between the propensity-matched non-DOAC and DOAC groups (46.94 ± 14.33 mL/m2 vs. 56.05 ± 19.31 mL/m2, respectively, *p* < 0.001). No significant difference was observed in the timing of echocardiography (2.11 ± 1.61 vs. 1.72 ± 1.09 months, *p* = 0.175).

### Comparison of CES patients with paroxysmal vs. permanent AF

Using the patient cohort of DOAC users and previously identified propensity- matched non-DOAC users, we evaluated the possible effect of AF burden on stroke severity. The median age of 147 paroxysmal AF patients was 79 years (27% male), while the 557 patients with permanent AF had a median age of 81 years (34% male). Significant differences were observed in age, CHA_2_DS_2_-VA score, GFR, arrival time, LAVI, LVEF, and NIHSS score at arrival (10.48 ± 7.01 vs. 9.18 ± 6.86, *p* = 0.09, favoring the paroxysmal AF group). However, an ANCOVA test adjusting for these parameters showed no significant difference in NIHSS score between groups (*p* = 0.67).

### Comparison of patients admitted to RAKUS and PSKUS

Combining the non-DOAC and DOAC cohorts, we found no significant differences in NIHSS at admission or echocardiographic parameters between hospitals. Further details are provided in Supplementary Table 2.

## Discussion

AF has become the most significant cardiac rhythm disorder as its prevalence is increasing in the aging population (1). Anticoagulation remains a cornerstone of preventive treatment in AF.

This study successfully validated the translation of epidemiological DOAC adherence data into real-world observations within acute stroke units. Our study highlights that patients with known AF who are not anticoagulated represent the primary demographic from CES. National Health Service prescription data correlates with these clinical findings, revealing that only 44.2% of Latvian AF patients achieve regular DOAC intake (adherence >80%) (10). Intensified patient education about the necessity of regular DOAC adherence is a straightforward opportunity to reduce CES burden.

Intensified education of primary care professionals also showed encouraging results. By comparing the stroke numbers from 2022 with those from 2024, we observed a 10.2% decrease in CES cases and a 19.1% decrease in AF patients who were not on anticoagulation therapy before stroke. Accordingly, there was a 15% increase in the number of prescribed anticoagulants (from 451,323 in 2022 to 519,136 prescriptions in 2024) as per unpublished data from the National Health Service. Furthermore, the number of AF patients with CES being on a subtherapeutic dose of warfarin has dwindled in this period. This is reflected by the unpublished data from the National Health Service, as the prescriptions of Vitamin K antagonists fell by 35.1% from 2022 to 2024. A further improvement of primary non-adherence would be achieved when the Latvian National Health Service compensates the DOACs already in patients with a CHA_2_DS_2_-VA score of ≥2 instead of the current CHA_2_DS_2_-VASc score ≥4. However, our data shows that CES patients not on DOAC therapy had a mean CHA_2_DS_2_-VA score of 4.3 ± 1.5 and a median score of 4.

Our data support the recently introduced “AF burden” paradigm, as only about 22% of stroke patients with known AF had the paroxysmal form (31). Once CES occurred, no significant difference in stroke severity between AF forms was observed.

In accordance with the recent study by Grosse et al. (20), we also observed a significant difference in stroke severity in patients on DOAC therapy (a 2.8-point lower NIHSS score, on average). This difference persisted even after rigorous propensity matching. The German multi-center cohort of AF patients with all stroke aetiologies observed a difference in NIHSS score between DOAC users and non-users of −2.5 points (20), while our study focused exclusively on cardioembolic aetiologies, which presented with higher baseline severity. Therefore, the reduction in stroke severity conferred by prior DOAC use should be interpreted in absolute, rather than relative, terms.

Notably, both patient groups exhibited severely enlarged left atria, yet DOAC users demonstrated a substantially higher LAVI (56.1 ± 19.3 vs. 47.2 ± 14.8 mL/m^2^ in non-users; *p* < 0.001). This structural discrepancy persisted through propensity matching. However, this result is primarily hypotheses-generating and larger echocardiography studies are required to confirm our results and, probably, finesse a cut-off value of LAVI as a risk factor.

PSKUS patients showed significantly lower NIHSS and mRS scores at discharge, but also significantly higher thrombectomy and thrombolysis rates. These inter-hospital differences likely reflect that PSKUS hosts the national interventional cardiology and neuroradiology center, with 24/7 thrombectomy availability. Despite these structural differences, baseline stroke severity (NIHSS at admission) and echocardiographic parameters were comparable, supporting the validity of pooling both cohorts for the primary analysis.

Although there was a strong female predominance (approximately 66%) throughout all patient groups, one must consider that a female-to-male ratio in the Latvian population above 65 is roughly 2:1, based on data from 2016 (32).

### Limitations

Although the two university hospitals in Riga serve the majority of the Latvian population (as primary and secondary medical facilities), this study does not represent data on the national level per se. Due to limited resources, we could not obtain data from much smaller regional primary and secondary medical facilities. Additionally, the study had a retrospective observational character. Therefore, not all information was obtained during the patient’s hospitalization, especially in those with severe (and fatal) stroke. We could not evaluate the impact of socioeconomic status on DOAC usage, arrival time, or stroke severity. In comparison of DOAC users vs. non-DOAC users, we excluded documented recurrent stroke cases to avoid possible confounding. However, no systematic follow-up has been conducted.

## Conclusion

While the non-adherence to regular DOAC therapy is a global problem, it is very important in Latvia, where 30.6% do not use DOACs and 55.8% (including those 30.6%) do not use DOACs regularly. Our study has focused specifically on CES patients with AF and identified non-DOAC users as the largest patient group. DOACs not only reduce the risk of CES, but their usage is also associated with reduced stroke severity. Despite preserved LVEF in both groups, DOAC users showed substantially greater LAVI, suggesting a structural component to thromboembolic risk that warrants further investigation.

## Data Availability

The raw data supporting the conclusions of this article will be made available by the authors, without undue reservation.
