# Supplementary material for: Real-world use and impact of direct oral anticoagulants among atrial fibrillation patients with cardioembolic stroke
Source: Front Neurol. 2026 Jun 18;17:1844341. doi: 10.3389/fneur.2026.1844341 (PMC13322816; doi:10.3389/fneur.2026.1844341)
Supplement: Supplementary file 1 [file Table_1.docx]

Supplementary Table 1. All baseline characteristics, treatments, and outcomes are stratified by ten-year age cohorts (< 60, 60 – 69, 70 – 79, 80 – 89, ≥ 90 years) for non-DOAC users among the enrolled CES patients with AF.

|  | <60 years | 60-69 years | 70-79 years | 80-89 years | ≥90 years | Total |
| --- | --- | --- | --- | --- | --- | --- |
| Total (n) | 38 | 124 | 310 | 479 | 109 | 1060 |
| Age (Mean ± SD) | 53.0 ± 5.5 | 65.6 ± 2.5 | 75.1 ± 2.8 | 84.3 ± 2.7 | 92.5 ± 2.2 | 79.1 ± 9.4 |
| Sex (F / M) | 14 / 24 | 50 / 74 | 178 / 132 | 361 / 118 | 93 / 16 | 696 / 364 |
| AF type (permanent / paroxysmal) | 23 / 15 | 95 / 29 | 227 / 83 | 381 / 98 | 94 / 15 | 820 / 240 |
| CHA2DS2-VA Score | 1.8 ± 1.4 | 3.2 ± 1.4 | 4.2 ± 1.4 | 4.6 ± 1.3 | 4.5 ± 1.2 | 4.2 ± 1.5 |
| Congestive heart failure | 22 | 63 | 181 | 306 | 83 | 655 |
| Art. hypertension | 24 | 102 | 261 | 418 | 92 | 897 |
| GFR (ml / min) | 80.3 ± 26.3 | 78.1 ± 24.4 | 72.9 ± 21.4 | 64.4 ± 20.8 | 60.2 ± 21.0 | 68.6 ± 22.4 |
| Diabetes mellitus | 3 | 31 | 62 | 85 | 11 | 192 |
| Prior stroke / TIA / TE | 7 | 42 | 100 | 159 | 30 | 338 |
| Vascular diseases | 6 | 30 | 90 | 133 | 26 | 285 |
| Arrival time period (1 / 2 / 3) | 20 / 0 / 18 | 62 / 16 / 46 | 155 / 42 / 111 | 213 / 38 / 228 | 39 / 12 / 56 | 489 / 108 / 459 |
| NIHSS at arrival | 10.0 ± 6.6 | 9.8 ± 6.4 | 10.7 ± 7.4 | 11.9 ± 7.2 | 15.3 ± 7.4 | 11.6 ± 7.3 |
| NIHSS at discharge | 4.3 ± 4.6 | 4.8 ± 4.2 | 6.0 ± 5.9 | 7.2 ± 6.5 | 9.1 ± 7.1 | 6.6 ± 6.2 |
| NIHSS difference | 4.8 ± 5.1 | 3.9 ± 4.5 | 3.5 ± 4.7 | 3.2 ± 4.2 | 3.7 ± 4.6 | 3.5 ± 4.5 |
| Thrombolysis | 13 | 37 | 99 | 136 | 30 | 315 |
| Thrombectomy | 6 | 15 | 38 | 45 | 3 | 107 |
| LAVI (ml/m2) | 46.9 ± 13.2 | 47.5 ± 19.7 | 45.0 ± 10.6 | 48.7 ± 15.9 | 50.0 ± 8.8 | 47.2 ± 14.8 |
| LVEF (%) | 53.2 ± 12.8 | 50.4 ± 11.0 | 51.1 ± 10.4 | 53.0 ± 10.1 | 50.4 ± 9.6 | 51.7 ± 10.5 |
| HAS-BLED Score | 0.8 ± 0.9 | 1.3 ± 0.9 | 1.7 ± 0.8 | 1.7 ± 0.7 | 1.7 ± 0.8 | 1.6 ± 0.8 |

All baseline characteristics, treatments, and outcomes are stratified by ten-year age cohorts (< 60, 60 – 69, 70 – 79, 80 – 89, ≥ 90 years) for DOAC users among the enrolled CES patients with AF.

| DOAC | <60 years | 60-69 years | 70-79 years | 80-89 years | ≥90 years | Total |
| --- | --- | --- | --- | --- | --- | --- |
| Total (n) | 13 | 45 | 87 | 180 | 27 | 352 |
| Age (Mean ± SD) | 54.8 ± 5.9 | 65.4 ± 2.6 | 74.7 ± 2.6 | 84.3 ± 2.6 | 92.0 ± 2.5 | 79.0 ± 9.2 |
| Sex (F / M) | 4 / 9 | 24 / 21 | 49 / 38 | 139 / 41 | 23 / 4 | 239 / 113 |
| AF type (permanent / paroxysmal) | 8 / 5 | 37 / 8 | 66 / 21 | 148 / 32 | 23 / 4 | 282 / 70 |
| CHA2DS2-VA Score | 2.2 ± 1.5 | 3.8 ± 1.6 | 4.5 ± 1.3 | 4.9 ± 1.2 | 4.8 ± 1.3 | 4.6 ± 1.4 |
| Congestive heart failure | 7 | 28 | 58 | 128 | 22 | 243 |
| Art. hypertension | 10 | 39 | 74 | 154 | 22 | 299 |
| GFR (ml / min) | 83.9 ± 17.5 | 75.7 ± 25.1 | 63.6 ± 20.4 | 62.3 ± 19.8 | 56.5 ± 23.2 | 64.7 ± 21.7 |
| Diabetes mellitus | 0 | 17 | 27 | 29 | 1 | 74 |
| Prior stroke / TIA / TE | 5 | 20 | 37 | 80 | 11 | 153 |
| Vascular diseases | 2 | 15 | 29 | 54 | 8 | 108 |
| Arrival time period (1 / 2 / 3) | 12 / 0 / 1 | 28 / 2 / 15 | 47 / 8 / 32 | 83 / 21 / 76 | 11 / 5 / 11 | 181 / 36 / 135 |
| NIHSS at arrival | 7.5 ± 5.8 | 9.2 ± 7.0 | 8.6 ± 6.6 | 9.2 ± 6.9 | 7.1 ± 6.1 | 8.8 ± 6.8 |
| NIHSS at discharge | 3.7 ± 4.6 | 4.9 ± 4.6 | 3.7 ± 4.6 | 6.2 ± 6.7 | 4.8 ± 5.0 | 5.3 ± 5.9 |
| NIHSS difference | 3.8 ± 3.9 | 3.3 ± 5.8 | 3.8 ± 4.3 | 2.5 ± 3.8 | 1.4 ± 2.2 | 2.9 ± 4.2 |
| Thrombolysis | 1 | 1 | 3 | 6 | 1 | 12 |
| Thrombectomy | 3 | 8 | 12 | 12 | 0 | 35 |
| LAVI (ml/m2) | 45.5 ± 19.7 | 61.2 ± 25.4 | 56.9 ± 20.6 | 55.2 ± 15.6 | 54.0 ± 6.0 | 56.1 ± 19.3 |
| LVEF (%) | 50.4 ± 14.6 | 48.7 ± 13.7 | 49.6 ± 12.1 | 54.9 ± 7.6 | 58.7 ± 11.0 | 52.0 ± 11.1 |
| HAS-BLED Score | 0.4 ± 0.7 | 1.2 ± 0.9 | 1.7 ± 0.8 | 1.7 ± 0.7 | 1.7 ± 0.9 | 1.6 ± 0.8 |

*(Abbreviations: AF, atrial fibrillation; CES, cardioembolic stroke; DOAC, direct oral anticoagulants; GFR, glomerular filtration rate; LAVI, left atrial volume index; LVEF, left ventricular ejection fraction; NIHSS, National Institutes of Health Stroke Scale; mRS, modified Rankin Scale; PSKUS, Paul Stradins Clinical University Hospital; RAKUS, Riga East University Hospital; TE, thromboembolic event; TIA, transient ischemic attack)*.

Supplementary Table 2. A comparison of the enrolled CES patients on DOACs and not using the DOACs, either admitted to PSKUS or RAKUS.

|  | Total (n) | PSKUS (n = 662) | RAKUS (n = 750) | p-value |
| --- | --- | --- | --- | --- |
| **Age** | **1412** | **78.1 ± 9.7** | **80.0 ± 9.0** | **< 0.001** |
| Sex | 1412 | 427 F / 235 M | 508 F / 242 M | 0.2 |
| AF type (parox. / perm.) | 1412 | 156 / 506 | 154 / 596 | 0.17 |
| **CHA_2_DS_2_-VA Score** | **1412** | **4.37 ± 1.52** | **4.22 ± 1.41** | **0.044** |
| **Congestive heart failure** | **1412** | **382** | **516** | **< 0.001** |
| **Art. hypertension** | **1412** | **593** | **603** | **< 0.001** |
| **GFR (ml / min)** | **1411** | **68.70 ± 21.72** | **66.68 ± 22.78** | **0.039** |
| Diabetes mellitus | 1412 | 126 | 140 | 0.860 |
| **Prior stroke / TIA / TE** | **1412** | **254** | **237** | **0.008** |
| **Vascular diseases** | **1412** | **227** | **166** | **< 0.001** |
| Arrival time period (1 / 2 / 3) | 1412 | 320 / 77 / 265 | 352 / 67 / 331 | 0.26 |
| NIHSS at arrival | 1408 | 11.24 ± 7.55 | 10.65 ± 7.01 | 0.231 |
| **NIHSS at discharge** | **1209** | **5.72 ± 5.85** | **6.64 ± 6.34** | **0.011** |
| **NIHSS difference** | **1209** | **4 ± 4.84** | **2.79 ± 3.9** | **< 0.001** |
| **Thrombolysis** | **1412** | **199** | **128** | **< 0.001** |
| **Thrombectomy** | **1412** | **122** | **20** | **< 0.001** |
| LAVI (ml/m2) | 357 | 50.71 ± 17.74 | 48.73 ± 15.24 | 0.419 |
| LVEF (%) | 357 | 51.68 ± 10.7 | 52.02 ± 10.7 | 0.804 |
| HAS-BLED Score | 1412 | 1.58 ± 0.82 | 1.62 ± 0.77 | 0.216 |
| mRS score at arrival | 1412 | 4.11 ± 1.1 | 4.05 ± 1.13 | 0.083 |
| **mRS score at discharge** | **1209** | **3.05 ± 1.49** | **3.32 ± 1.42** | **0.002** |
| **mRS score difference** | **1209** | **0.9 ± 1.19** | **0.63 ± 0.96** | **< 0.001** |

The RAKUS patients were significantly older, had congestive heart failure more frequently, while the PSKUS patients had a higher number of previous thromboembolic events and vascular diseases, thus also a significantly higher CHA2DS2-VA score. Patients in PSKUS received thrombolysis and thrombectomy more frequently. There was no significant difference in NIHSS score at admission and echocardiographic parameters between patients admitted to these hospitals.

*(Abbreviations: AF, atrial fibrillation; GFR, glomerular filtration rate; LAVI, left atrial volume index; LVEF, left ventricular ejection fraction; NIHSS, National Institutes of Health Stroke Scale; mRS, modified Rankin Scale; PSKUS, Paul Stradins Clinical University Hospital; RAKUS, Riga East University Hospital; TE, thromboembolic event; TIA, transient ischemic attack)*.
